# Inhibition of Advanced Glycation End Products: A Nexus of Chicken Hyperglycemia and Inflammation Absence

**DOI:** 10.3390/biology14121657

**Published:** 2025-11-24

**Authors:** Shuwen Luo, Jinlu Liu, Yujiao Guo, Wang Gu, Mingfeng Wang, Yu Zhang, Guohong Chen, Qi Xu

**Affiliations:** 1 College of Animal Science and Technology, Yangzhou University, Yangzhou 225009, China; 2 Key Laboratory for Evaluation and Utilization of Livestock and Poultry Resources (Poultry), Ministry of Agriculture and Rural Affairs, Beijing 100176, China

**Keywords:** advanced glycation end products, fructosamine, methylglyoxal, free amino acid, inflammation

## Abstract

Poultry have higher blood glucose concentrations, which are 1.5–2 times those of their mammalian counterparts with equivalent body mass, yet do not show any adverse effects. However, the underlying mediators that inhibit systemic inflammation under hyperglycemia are unclear. In this study, chickens and rats were chosen as representatives of poultry and mammals, and the physiological differences in blood glucose regulation between them were investigated under control conditions and streptozotocin (STZ)-induced hyperglycemia, respectively, to explore the internal causes of hyperglycemia without symptoms in poultry. Firstly, the fasting blood glucose (FBG) and glucagon concentrations increased significantly (*p* < 0.01) and the insulin concentrations decreased when chickens and rats were treated with STZ (*p* < 0.001). STZ injections in rats resulted in higher oral glucose tolerance test (OGTT) and intraperitoneal insulin tolerance test (IPITT) levels (*p* < 0.001), but there was no significant difference in chickens. In addition, the body weight development of STZ-inducted rats was retarded, while it was not the case for chickens receiving STZ. Secondly, high glucose metabolism products, including advanced glycation end products (AGEs) were detected in STZ-inducted rats and chickens. The AGEs concentration in the rats receiving STZ was significantly higher than that in control group rats (*p* < 0.001); however, there was no significant difference in chickens. Also, the concentrations of free amino acids inhibiting AGEs were further explored, and higher concentrations of taurine, leucine, and lysine were observed in chickens than those observed in rats (*p* < 0.05). Moreover, the concentrations were decreased significantly in STZ-treated chickens (*p* < 0.05). Finally, the inflammation in tissues vulnerable to high blood glucose was examined, and it was found that there were significantly increased mRNA and protein expression levels of inflammatory factors such as nuclear factor-κB (NF-κB) and interleukin-1β (IL-1β) in STZ-injected rats (*p* < 0.001), while there was no obvious effect in STZ-induced chickens. The results revealed the damage and inflammation resulting from STZ-induced hyperglycemia in chickens were significantly lower compared to rats. This may be attributed to the high concentrations of free amino acids in chickens, which inhibit AGE formation by functioning as carbonyl scavengers. This study elucidates the underlying causes of the absence of inflammation in chickens compared to rats under hyperglycemic conditions, offering new insights for controlling diabetic complications.

## Simple Summary

Poultry have unique traits in maintaining glucose homeostasis. Their blood glucose concentrations are 1.5–2 times higher than those observed in mammals, yet with no inflammation. This study investigated why poultry are less prone to inflammation under hyperglycemic conditions using chickens and rats as models. The damage and inflammation resulting from STZ-induced hyperglycemia in chickens were significantly lower than those in rats, and the levels of free amino acids in chickens were significantly higher than those in rats. These free amino acids may inhibit the formation of AGEs by trapping carbonyl groups, thereby suppressing the generation of inflammation. These results not only shed light on the anti-inflammatory effect of chronic hyperglycemia in chickens but also offer new targets for developing therapeutic agents to regulate hyperglycemia.

## 1. Introduction

Poultry exhibit unique glucose homeostasis. Firstly, blood glucose concentrations are twofold higher than those observed in mammals [1,2]. Secondly, the chronically elevated concentrations of blood glucose (chronic hyperglycemia) have not caused any adverse effects even in a fasted state, including diabetes mellitus and its associated complications [3,4]. It has been preliminarily proven that glucose homeostasis of poultry was correlated with the markedly higher standard metabolic rate and body temperature of birds compared to mammals [5,6]. Studies have shown that hummingbirds and bats can use hyperglycemia to provide fuel for flight [7,8]. However, these conventions in mammals dictate that these parameters are expected to result in an increased level of tissue damage, such as retinopathy, nephropathy, neuropathy, microvascular damage, and macrovascular damage [9,10]. Previous studies have focused only on physiological-level correlates (e.g., metabolic rate and body temperature) and failed to explore the underlying causes of poultry’s resistance to hyperglycemic complications. Chickens, as a type of poultry, have lost the ability to fly and migrate, but still maintain high glucose concentration. To the best of our knowledge, the underlying mediators that regulate blood glucose homeostasis to prevent diabetic complications under chronic hyperglycemia in chickens have yet to be clarified.

Maillard reaction (MR) is one of the main factors contributing to diabetic complications [11] and is a reaction involving the condensation between a carbonyl group of reducing sugars, aldehydes, or ketones, and an amino group of amino acids, proteins or any nitrogenous compound [12]. MR is a chemical procedure that involves three steps through which the initial products are identified as the slow formation of Schiff bases that via rearrangement reaction could form Amadori products, which undergo further reactions to produce irreversible advanced glycation end products (AGEs) [13,14]. Fructosamine (FA) is a product of the Schiff base reaction in the early stage of the Maillard reaction, referring to all ketoamine linkages that result from the glycation of serum proteins [15,16]. While methylglyoxal (MG) is an intermediate product of the Maillard reaction, it has two adjacent carbonyl groups that can directly form AGEs [17,18]. AGEs are deleterious molecules and they transduce inflammatory and fibrotic signals in immune-related cells to induce somatic cell damage, and lastly, tissue fibrosis in various tissue types and organs [19,20]. Accordingly, understanding the generation and development of AGEs is of crucial importance in the research of the underlying mechanisms of diabetic complications.

Streptozotocin (STZ) is an antibiotic that raises blood glucose by destructing pancreatic islet β-cells and is widely used experimentally to produce a model of diabetes mellitus (DM) [21]. Also, previous research suggests that blood glucose concentrations in chickens and rats could be increased by intraperitoneal injection of STZ [22,23]. However, the physiological differences in blood glucose regulation between them have been rarely reported. Additionally, studies have indicated that the breakthrough of using poultry as a non-pathological model for T2DM lies in their low concentration of AGEs, which may be attributed to the anti-glycosylation properties of poultry, but there is currently no data to support this claim [24,25]. To clarify the potential link between poultry physiology and AGE inhibition, prior studies have provided critical evidence for the anti-glycation role of free amino acids. Specifically, it has been proven that many free amino acids inhibit the formation of AGEs, including histidine, alanine, taurine, and arginine [26,27]. In addition to any potential hypoglycemic effects, their more important function is to bind to the carbonyl groups of glucose to competitively inhibit the carbonyl–amine reactions [28,29]. Thus, free amino acids are also known as “carbonyl scavengers” for their ability to intercept glucose-derived carbonyl groups and block AGE formation, thereby inhibiting the MR.

Therefore, in this study, chickens and rats were chosen as representatives of poultry and mammals, respectively, to investigate the differences in substances such as insulin, AGEs, and free amino acids in the blood under normal conditions and STZ-induced hyperglycemia. The objective of this study was to explore the internal causes of why mammals develop hyperglycemic complications while poultry show no clinical symptoms, and specifically to investigate whether the reduction in free amino acid concentration inhibits the occurrence of MRs by comparing blood glucose regulation between chickens and rats under hyperglycemia. We hypothesize that higher concentrations of free amino acids in chicken plasma reduce the formation of AGEs under hyperglycemic conditions. In contrast, the insufficiency of free amino acids in rat plasma fails to prevent the occurrence of MR and the accumulation of AGEs, ultimately contributing to the development of hyperglycemic complications. This difference is expected to be the key internal reason for the absence of clinical symptoms in hyperglycemic poultry. The results not only shed light on the anti-inflammatory effect of chronic hyperglycemia in chickens but also offer new targets for developing therapeutic agents to regulate hyperglycemia.

## 2. Materials and Methods

### 2.1. Ethics Statement

Animal care and handling protocols were approved by the Yangzhou University Ethics Committee for Animal Experiments (Permit Number: 2023004742). All procedures adhered to the “Regulations on Laboratory Animal Management” (Yangzhou University, 2012) and the “Standards for Experimental Practices” (Jiangsu, China, 2008).

### 2.2. Experimental Animals

Thirty 20-day-old male Ross 308 chickens (body weight: 0.7–1 kg) were sourced from the Hongwei Chicken Farm (Suqian, China), and thirty male Sprague Dawley (SD) rats at 4 weeks of age (body weights: 70–100 g) were obtained from the Animal Center of Yangzhou University (Yangzhou, China). Thirty animals per species were used to account for potential losses (e.g., health issues and accidental injury) and align with the animal facility’s capacity, with only healthy individuals included per preset screening criteria. The healthy chickens were housed in an 18.5-square-foot area with 6 wire cages, each containing 4–6 birds, and were kept under a 16 h light and 8 h dark cycle at a temperature range of 22–26 °C for 7 days. The rats were housed in a 20-square-foot room with 9 cages, each containing 3–4 rats and were maintained under a 12 h light and 12 h dark cycle, with room temperature set between 23 and 26 °C. The composition of the basal diets and nutrient levels for chickens and rats is presented in Table 1 and Table 2. There was no additional glucose added to the daily diets consumed by the experimental animals.

### 2.3. Hyperglycemic Animal Model

After 7 days of acclimation, thirty chickens and thirty rats were fasted for 8 h with ad libitum access to water before the experiment. These animals were randomized into two groups, respectively. Fifteen chickens and fifteen rats received intraperitoneal injections of streptozotocin (STZ; 70 mg/kg in 0.1 M citrate-buffered saline, pH 4.5) to induce diabetes (572201, Sigma-Aldrich, St. Louis, MO, USA), while the remaining animals were injected with PBS (1 mL) as a control. The injection was provided once every three days, twice at the beginning of the trial, totaling six days. Subsequently, the STZ-treated chickens and rats fasted for 12 h prior to blood glucose measurements. The fast blood glucose (FBG) concentrations were measured with an ACCU-CHEKA Performa glucometer (Roche Diagnostics GmbH, Mannheim, Germany). Chickens with fasting glucose concentrations > 14 mmol/L were classified as hyperglycemic [22]. Rats with fasting glucose concentrations > 12 mmol/L were considered hyperglycemic [30].

### 2.4. Oral Glucose Tolerance Tests and Intraperitoneal Insulin Tolerance Tests

For oral glucose tolerance tests (OGTTs), chickens and rats were fasted overnight without water restriction on day 14, after which glucose (2 g/kg body weight, BW) was administered orally. Blood glucose levels were measured at 0, 30, 60, 90, and 120 min post-administration using an ACCU-CHEK Performa glucometer.

For intraperitoneal insulin tolerance tests (IPITTs), chickens and rats were fasted overnight without water restriction on day 17. Subcutaneous insulin injection (2 IU/kg BW) was administered, and blood glucose concentrations were determined at 0, 30, 60, 90, and 120 min post-injection using the same glucometer. The homeostatic model assessment for insulin resistance (HOMA-IR) index was calculated using the following formula: HOMA-IR = (FBG [mmol/L] × FINS [units/L])/22.5 [31], where FBG denotes fasting blood glucose and FINS represents fasting insulin.

### 2.5. Sample Collection

All animals were monitored daily for any health-related problems. The experimental study lasted for 21 days after successful induction with STZ. At the end of the experiment, six chickens and six rats per group were selected as replicates using a random sequence generated via Microsoft Excel (Microsoft Corp., Redmond, WA, USA), with priority given to healthy individuals free of specific symptoms; blood collection was then performed at the Animal Center of Yangzhou University. The serum was separated from the blood samples by centrifuging at 3000× *g* for 10 min at 4 °C. Following labeling, the serum samples were rapidly frozen in liquid nitrogen for 10 min and then cryostored at −80 °C for subsequent experimental use. After the treatment ended, all chickens and rats were anesthetized using 10% chloral hydrate and then humanely sacrificed. The kidney, liver, heart, muscle (pectoral muscle), and pancreas were collected sequentially. The kidney and liver were weighed after removing related contents and adherent substances. Organ indices were determined using the following formula: [organ weight (g)/live body weight (g)] × 100%. Then, different organs were promptly collected into cryotubes, frozen in liquid nitrogen, and stored at −80 °C for real-time RT-qPCR analysis. Additionally, the pancreas was also harvested for Hematoxylin–Eosin (H&E) staining.

### 2.6. Determination of Serum Hormone Concentrations by ELISA

Serum concentrations of insulin, glucagon, fructosamine (FA), and advanced glycation end products (AGEs) in chickens and rats were detected by ELISA, respectively, following the protocols from commercial kits (for chickens, MM-60632O2, MM-60623O2, MM-60621O2, and MM-60615O2; for rats, MM-1049R2, MM-8272R2, MM-21260R2, and MM-0270R2, Jiangsu Kete Biotechnology Co., Ltd., Yancheng, China). The concentrations of nuclear factor-κB phosphorylated p65 (NF-κB p-p65), interleukin-1β (IL-1β), and interleukin-10 (IL-10) were also measured by ELISA using commercially available kits (for chickens: ml002789, ml059835, and ml059830; for rats: ml259671, ml037361, and ml037371; Shanghai Enzyme-linked Biotechnology Co., Ltd., Shanghai, China). Briefly, 50 µL of the prepared sample, standards, and biotin antigen were added to each well and incubated for 30 min at 37 °C. After washing the plate 5 times, 50 µL of horseradish peroxidase conjugate reagent was added, followed by another 30 min incubation at 37 °C. The plate was washed again 5 times, and 50 µL of chromogen solution A and B was added, with further incubation for 30 min at 37 °C. Lastly, 50 µL of stop solution was added, and optical density (OD) values were recorded for calculation within 10 min.

### 2.7. Blood Biochemical Analysis

After blood collection, centrifugation, and storage, the serum biochemical indicators were determined using a fully automatic biochemical analyzer (AU480, Beckman Coulter K.K, Tokyo, Japan). Serum alanine aminotransferase (ALT), aspartate aminotransferase (AST), the AST/ALT, alkaline phosphatase (ALP), total protein (TP), albumin (ALB), globulin (GLOB), and ALB/GLOB were determined. The blood lipid indicators measured were triglyceride (TG), total cholesterol (TC), low density lipoprotein cholesterol (LDL-C), high density lipoprotein cholesterol (HDL-C).

### 2.8. RT-qPCR

Total RNA was isolated from the kidney, liver, heart, muscle, and pancreas. Primers were designed via Primer 5.0 software (Premier Biosoft International, Palo Alto, CA, USA) and custom-synthesized by Tsingke Biotechnology Co., Ltd. (Nanjing, China) to detect the expression levels of the following target genes: nuclear factor kappa B (NF-κB), interleukin-1 beta (IL-1β), tumor necrosis factor alpha (TNFα), monocyte chemoattractant protein-1 (MCP1), and interleukin-10 (IL-10) (Table 3). The reaction was performed using 2× Q3 SYBR qPCR Master Mix (Universal) (22204, Tolo Biotech Co., Ltd., Shanghai, China). For each experimental group, six samples per tissue were selected, with three technical replicates per sample. Data were normalized against the housekeeping gene β-actin, and relative abundance was calculated by applying the comparative CT method 2−∆∆CT.

### 2.9. Western Blot Analysis

Tissues were lysed with RIPA laced with proteinase inhibitors for 30 min, and the protein concentration was measured using the BCA assay (P0010S, Beyotime Biotechnology, Shanghai, China) following the manufacturer’s instructions. The protein samples were denatured at 100 °C for 7 min containing 5× loading buffer (P0285, Beyotime Biotechnology, Shanghai, China). The 12% sodium dodecyl-sulfate polyacrylamide gel electrophoresis (SDS-PAGE) gel was prepared using an SDS-PAGE Gel Kit (P1200, Solarbio Science & Technology Co., Ltd., Beijing, China). Then, 20 μg of total protein was subjected to 12% SDS-PAGE, which was performed on ice using an electrophoresis system (PowerPac HC, Bio-Rad, Hercules, CA, USA). The electrophoresis parameters were set as follows: 90 V for 20 min for the stacking gel and 120 V for 40 min for the resolving gel. After electrophoresis, proteins were transferred onto 0.45 μm polyvinylidene fluoride (PVDF) membranes, which were incubated in 5% bovine serum albumin-blocking solution for 1 h at room temperature.

To detect protein markers of inflammatory factors, the membranes were incubated with primary antibodies including rabbit anti-NF-κB phosphorylated p65 (1:1000, AP1294, ABclonal Technology Co., Ltd., Wuhan, China), NF-κB total p65 (1:1000, A2547, ABclonal Technology Co., Ltd., Wuhan, China), IL1β (1:1000, A16288, ABclonal Technology Co., Ltd., Wuhan, China), and β-actin (1:2000, CW0097, CWBIO Technology Co., Ltd., Taizhou, China) at 4 °C overnight. After the excess primary antibodies on the PVDF membranes were cleaned, the membranes were incubated with secondary goat anti-rabbit antibodies which conjugated with horseradish peroxidase (HRP) (1:7500, CW0103S, CWBIO Technology Co., Ltd., Taizhou, China). After the excess secondary antibodies on the PVDF membranes were cleaned, the membranes were incubated with a chemiluminescence detection kit (P10100, NCM Biotech Co., Ltd., Soochow, China) following the manufacturer’s instructions and were detected with a chemiluminescence detector (Tanon5200, Shanghai, China).

### 2.10. H&E Staining and Masson Staining

After fixation, the pancreas samples were embedded in paraffin for 24 h first and then sectioned into 5 µm-thick sections using a microtome (RM2016, Leica Biosystems, Wetzlar, Germany). The sections were subjected to H&E staining (GP1031, Wuhan Servicebio Biotechnology Co., Ltd., Wuhan, China) for histological inspection. Histological images were captured using a micro-image analysis system (CaseViewer 2.4, 3DHISTECH Ltd., Budapest, Hungary).

### 2.11. Liquid Chromatography–Mass Spectrometry (LCMS)

AGILENT 1290–6470 liquid chromatography–mass spectrometry (Agilent Technologies Inc., Santa Clara, CA, USA) was used to obtain the detailed mass spectra of methylglyoxal (MG), taurine and free amino acids in the serum. The serum was filtered with a 0.22 µm nylon filter, and the sample was injected into the analytical column. The LCMS conditions for MG were as follows. Chromatographic column: Agilent C18 column (2.1 mm × 100 mm, 1.8 μm); column temperature: 35 °C; flow rate: 0.3 mL/min; acquisition mode: ESI+; parent ion: 195; daughter ions: 127, 115; injection volume: 2 μL; mobile phase: mobile phase A: 0.1% formic acid aqueous solution; mobile phase B: acetonitrile. The mobile phase gradient is shown in Table 4.

The LCMS conditions for 17 free amino acids were as follows: chromatographic column: C18 SHISEIDO column (4.6 mm × 250 mm × 5 μm, Shiseido Co., Ltd., Tokyo, Japan); detector: FLD detector; column temperature: 40 °C; injection volume: 10 μL; wavelength: 254 nm. Mobile phases: Mobile phase A: a mixture of 0.1 mol/L anhydrous sodium acetate and acetonitrile at a ratio of 97:3. After mixing, the pH was adjusted to 6.5 (prepared by dissolving 31.815 g of sodium acetate in 3880 mL of water and then adding 120 mL of acetonitrile). Mobile phase B: a mixture of acetonitrile and water at a ratio of 80:20. The mobile phase gradient is shown in Table 5. The LCMS unit was directly connected with Agilent Technologies version acquisition method info for the detailed analysis and mass fragmentation was identified by using a spectrum database for organic compounds.

### 2.12. Statistical Analysis

SPSS software (version 25.0, IBM Corp, Armonk, NY, USA) was used for statistical analysis. Each experiment was performed six times, and the data were expressed as mean ± SE. Prior to data analysis, the normality of the data was verified by the Shapiro–Wilk test, and the homogeneity of variances was confirmed by the Levene test. For comparisons involving two independent groups, an unpaired Student’s *t*-test was used. To investigate the effects of species (chicken/rat) and treatment (CON/STZ) and their interaction, a two-way ANOVA was conducted. Where a significant interaction was found, simple effects analysis was performed. Statistical significance was set at *p* < 0.05. GraphPad Prism 10.1 (GraphPad Software, San Diego, CA, USA) was used for processing figures.

## 3. Results

### 3.1. Effect of STZ on Blood Glucose Control and Insulin Sensitivity

**Figure 1:**
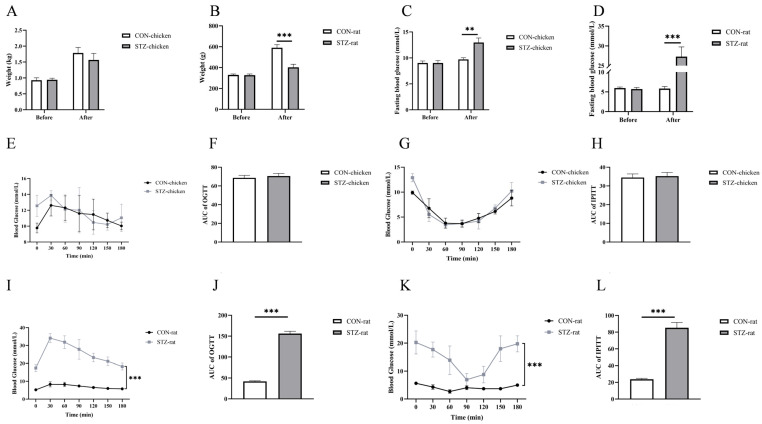
Effects of streptozotocin (STZ) treatment on body weight, blood glucose, and insulin sensitivity of chickens and rats. (**A**,**B**) The body weight of each group of chickens and rats was determined before and after STZ treatment. (**C**,**D**) Blood glucose concentrations of each group of chickens and rats were taken after fasting for 8 h determined before and after STZ treatment. (**E**) Individual glucose tolerance of chickens was assessed by the oral glucose tolerant test (OGTT). (**F**) The area under the curve for chickens’ OGTT. (**G**) Individual insulin tolerance of chickens was evaluated by intraperitoneal insulin tolerant test (IPITT). (**H**) The area under the curve for chickens’ IPITT. (**I**) Individual glucose tolerance of rats was assessed by OGTT. (**J**) The area under the curve for rats’ OGTT. (**K**) Individual insulin tolerance of rats was evaluated by the intraperitoneal insulin tolerance test (IPITT). (**L**) The area under the curve for rats’ IPITT. CON-chicken, control group chicken; STZ-chicken, STZ-treated group chicken; CON-rat, control group rat; STZ-rat, STZ-treated group rat. Data are expressed as the means ± SE. Data were compared by unpaired *t*-test. N = 6. ***, *p* < 0.001; **, *p* < 0.01.

To investigate the induction effect of streptozotocin (STZ) on chickens and rats, the weight, fast blood glucose (FBG) concentrations, and glucose and insulin tolerance of chickens and rats before and after STZ treatment were evaluated. Compared with the control group, there was no significant difference in the weight of chickens in the STZ-induced group (Figure 1A), while the weight of rats’ decreased significantly (*p* < 0.001; Figure 1B). For blood glucose concentrations, STZ treatment significantly increased the fasting blood glucose concentration of chickens and rats (*p* < 0.05; Figure 1C,D).

The glucose and insulin tolerance results showed that STZ induction had no significant effect on glucose tolerance and insulin sensitivity in chickens (*p* > 0.05; Figure 1E–H). However, it significantly disordered glucose and insulin tolerance in rats (*p* < 0.0001; Figure 1I–L).

### 3.2. Effect of STZ on Insulin and Glucagon Concentration

**Figure 2:**
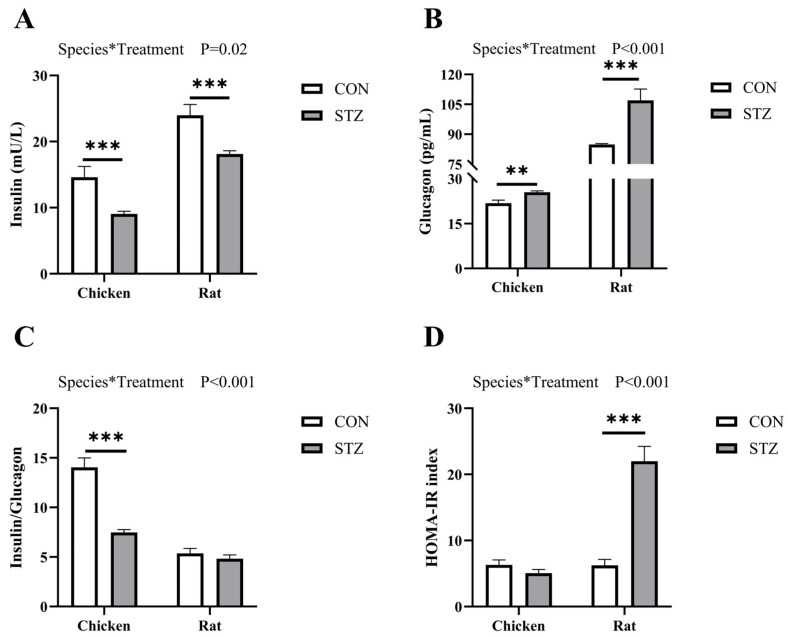
Effects of streptozotocin (STZ) treatment on insulin and glucagon level of chickens and rats. (**A**) The insulin concentration of each group of chickens and rats was determined before and after STZ treatment. (**B**) Glucagon level of each group of chickens and rats was determined before and after STZ treatment. (**C**) The insulin/glucagon index of chickens and rats. (**D**) The homeostatic model assessment for insulin resistance (HOMA-IR) index of chickens and rats. CON, control group; STZ, STZ-treated group. Data are expressed as the means ± SE. Two-way ANOVA was used to analyze species*treatment effects. The asterisk (*) denotes a significant interaction between species and treatment. N = 6. ***, *p* < 0.001; **, *p* < 0.01.

After 21 days of treatment with STZ, serum insulin and glucagon concentrations were measured to assess insulin resistance in all animals. Two-way ANOVA revealed a significant interaction between species and treatment on the concentrations of insulin and glucagon (*p* < 0.05, Figure 2). As shown, the insulin concentration in both chickens and rats decreased significantly (*p* < 0.001; Figure 2A), while glucagon increased significantly (Figure 2B). The ratio of insulin to glucagon in chickens decreased significantly after STZ induction (*p* < 0.001; Figure 2C). Although there was a downward trend in rats, there was no significant difference. The HOMA-IR index further indicated that rats developed significant insulin resistance (*p* < 0.001; Figure 2D).

### 3.3. Effect of STZ on Fructosamine, Methylglyoxal, and Advanced Glycation End Products Concentration

**Figure 3:**
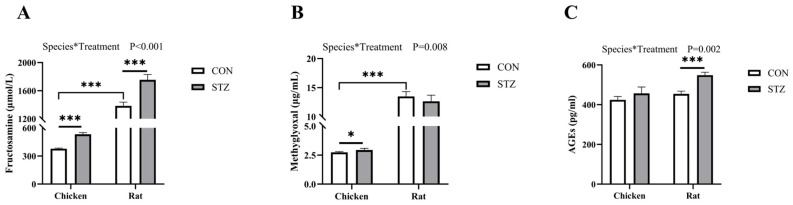
Effects of streptozotocin (STZ) treatment on fructosamine (FA), methylglyoxal (MG), and advanced glycation end product (AGE) concentrations of chickens and rats. (**A**) The FA concentration of each group of chickens and rats was determined before and after STZ treatment by ELISA. (**B**) The MG concentration of each group of chickens and rats was determined before and after STZ treatment by liquid chromatography–mass spectrometry (LCMS). (**C**) The AGE concentration of chickens and rats was determined before and after STZ treatment by ELISA. CON, control group; STZ, STZ-treated group. Data are expressed as the means ± SE. Two-way ANOVA was used to analyze species*treatment effects. The asterisk (*) denotes a significant interaction between species and treatment. N = 6. ***, *p* < 0.001; *, *p* < 0.05.

To investigate the role of STZ in blood glucose regulation, we first detected the content of fructosamine (FA) and methylglyoxal (MG) in the blood of chickens and rats. The FA level of chickens and rats was detected by ELISA. Two-way ANOVA revealed a significant interaction between species and treatment on the products of the MR (*p* < 0.01, Figure 3). The results showed that the FA concentrations of chickens and rats were significantly increased after STZ induction (*p* < 0.001; Figure 3A). The MG of chickens and rats was determined by liquid chromatography–mass spectrometry (LCMS). The detection results showed that the methylglyoxal concentrations of chickens were significantly increased after STZ induction (*p* < 0.05; Figure 3B), and there was no significant difference before and after induction in rats. However, before STZ induction, the concentrations of FA and MG in rats were significantly higher than those in chickens (*p* < 0.001; Figure 3A,B).

Given the fact that FA and MG are upregulated in chickens and rats treated with STZ, we speculated that the glucose Maillard reaction is mobilized. Next, we detected the concentration of advanced glycation end products (AGEs) in chickens and rats before and after STZ treatment. The results determined by ELISA showed that STZ induction increased the concentration of AGEs in chickens and rats, but there was no significant difference in chickens (Figure 3C). Among them, the difference in rats is highly significant (*p* < 0.001).

### 3.4. Effect of STZ on Blood Biochemical Parameters

Blood chemistry, measured through biochemical parameters, is a window into the chickens’ and rats’ health. These parameters reflect the chickens’ and rats’ nutritional status, physiological functions, and underlying diseases. Blood chemistry can be an effective tool for understanding how STZ treatment affects the health of chickens and rats. The results showed that there were significant differences in the blood biochemical indices between chickens and rats, whether STZ was induced or not. Except for a significant decrease in the GLO level (Table 6), STZ induction did not exert a significant effect on the liver function and blood lipid indexes of chickens. However, it significantly affected the liver function of rats (*p* < 0.05; Table 6 and Table 7). Among them, ALT, ALP, and GLO were significantly increased, while AST, ALB, and TP were significantly decreased. In addition, STZ induction significantly reduced the blood triglyceride concentration in rats, which also indirectly suggested that the liver function was damaged.

### 3.5. Effect of STZ on Organ Development and Damage

**Figure 4:**
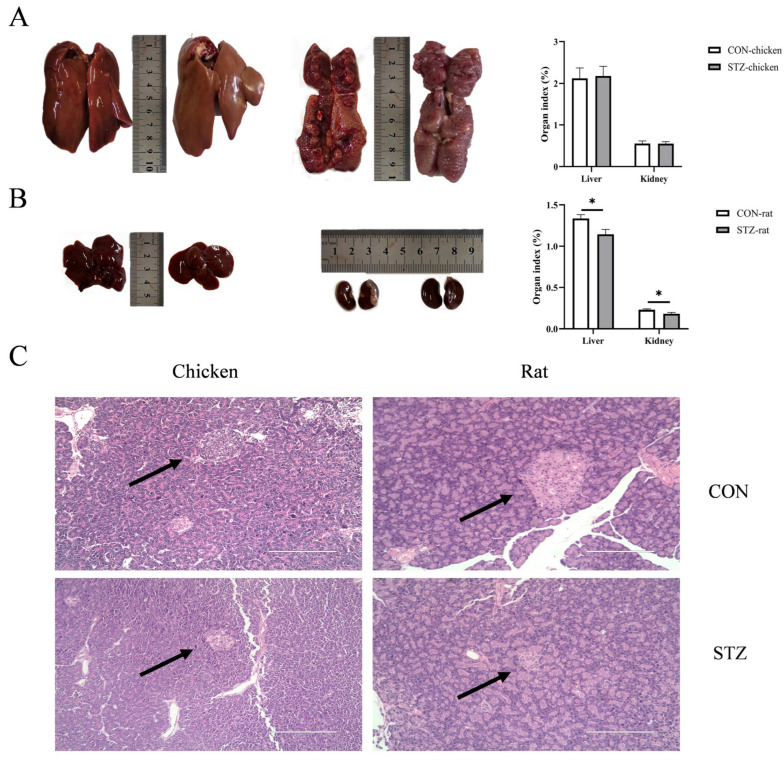
Effects of streptozotocin (STZ) treatment on organ development and damage of chickens and rats. (**A**,**B**) The liver and kidney organ indexes of each group of chickens and rats were determined before and after STZ treatment. Data are expressed as the means ± SE. Data were compared by an unpaired *t*-test. N = 6. *, *p* < 0.05. (**C**) Pancreatic histology was studied in serial 5 µm hematoxylin/eosin-stained (H-E) sections, which were observed under light microscopy with arrows highlighting pancreatic islets structures (Scale bar = 100 µm). Notably, for spherical spatial structures such as pancreatic islets of Langerhans, the size observed in microscopic sections depends on the sectioning position: an inherently large islet may appear small if sectioned near its edge, and vice versa. To minimize this variation, we specifically focused on sections passing through the central region of islets during observation and quantification. CON-chicken, control group chicken; STZ-chicken, STZ-treated group chicken; CON-rat, control group rat; STZ-rat, STZ-treated group rat.

To determine the effects of the glucose Maillard reaction mobilization by STZ on the organ development and damage of chickens and rats, the relevant parameters of target organs were measured. After being induced by STZ for 21 days, there was no significant difference in the weight of chickens (*p* > 0.05; Figure 1A). However, rats exhibited significant emaciation (*p* < 0.001; Figure 1B). Moreover, compared with the control group, the organ indices of liver and kidney of rats decreased in the STZ group (*p* < 0.05; Figure 4A,B). These two sets of indicator results indicate that STZ-induced rats suffered from delayed organ development. In contrast, there was no significant effect on the organ development of chickens. Notably, anatomically, domestic chicken kidneys are characterized by three lobes located posterior to the lungs and along the spine. Although the chicken kidney image in Figure 4A only captures a partial segment due to limitations during sample collection, the organ index data and subsequent histological analysis still robustly demonstrate that streptozotocin (STZ) induction did not cause notable morphological changes in chicken. In addition, for pancreatic histology, the pancreatic islets of chickens and rats induced by STZ appeared with an irregular morphology and a reduction in the area of the islets. In particular, the islets of rats showed obvious atrophy (Figure 4C).

### 3.6. Effect of STZ on Tissue Inflammation Expression

**Figure 5:**
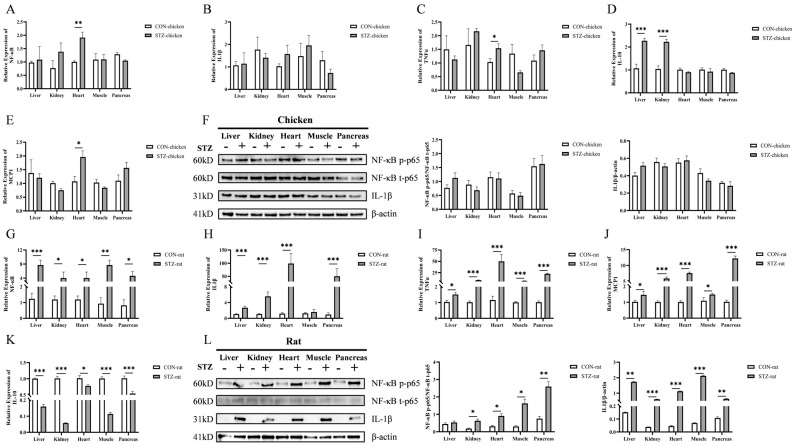
Effects of streptozotocin (STZ) on expression of inflammatory markers in tissues of chickens and rats. (**A**) The relative expression levels of NF-κB mRNA in chicken tissues. (**B**) The relative expression levels of IL-1β mRNA in chicken tissues. (**C**) The relative expression levels of TNFα mRNA in chicken tissues. (**D**) The relative expression levels of MCP1 mRNA in chicken tissues. (**E**) The relative expression levels of IL-10 mRNA in chicken tissues. All markers were determined by RT-qPCR before and after STZ treatment. N = 6. (**F**) The protein levels of p-NF-κB p65, NF-κB p65, and IL-1β in chicken tissues before and after STZ treatment were determined by Western blot. (**G**) The relative expression levels of NF-κB mRNA in rat tissues. (**H**) The relative expression levels of IL-1β mRNA in rat tissues. (**I**) The relative expression levels of TNFα mRNA in rat tissues. (**J**) The relative expression levels of MCP1 mRNA in rat tissues. (**K**) The relative expression levels of IL-10 mRNA in rat tissues. All markers were determined by RT-qPCR before and after STZ treatment. N = 6. All markers were determined by RT-qPCR before and after STZ treatment. CON-chicken, control group chicken; STZ-chicken, STZ-treated group chicken; CON-rat, control group rat; STZ-rat, STZ-treated group rat. Data are expressed as the means ± SE. N = 6. (**L**) The protein levels of p-NF-κB p65, NF-κB p65 and IL-1β in rat tissues before and after STZ treatment were determined by Western blot. Data were compared by an unpaired *t*-test. ***, *p* < 0.001; **, *p* < 0.01; *, *p* < 0.05.

Liver, kidney, heart, muscle, and pancreatic tissues are key target organs of glucose metabolism; the expression of inflammatory factors in these tissues was detected. As shown, in various target organs of chickens, excluding the heart, there was no significant difference in the mRNA expression of nuclear factor-κB (NF-κB), interleukin-1β (IL-1β), tumor necrosis factor α (TNFα), and monocyte chemoattractant protein 1 (MCP1) regardless of STZ induction (Figure 5A–D). In contrast, in the chicken heart, STZ induction led to a significant increase in the mRNA expression of NF-κB, TNFα, and MCP1, while in rats, the expression of NF-κB, IL-1β, TNFα, and MCP1 was significantly increased after STZ treatment (Figure 5G–J). For the anti-inflammatory factor interleukin-10 (IL-10), levels in the liver and kidney were significantly increased in STZ-induced hyperglycemic chickens (Figure 5E), whereas they were significantly decreased in all organs of STZ-induced hyperglycemic rats (Figure 5K). The Western blotting (WB) results showed that STZ treatment had no significant effect on the protein expression of NF-κB p-p65 and IL-1β in various tissues of chickens (Figure 5F and Figure S1). However, in rats, STZ induction exerted organ-specific effects on the protein expression of NF-κB p-p65: it had no significant effect on NF-κB p-p65 in the liver, but significantly activated this protein in the kidney, heart, muscle, and pancreas; meanwhile, STZ also significantly increased the protein expression of IL-1β across various organs (Figure 5L and Figure S2). Figures S1 and S2 provide the original uncropped WB blots for chickens and rats, respectively, to support the cropped results presented in Figure 5F,L.

In addition, the concentrations of serum NF-κB p-p65, IL-1β, and IL-10 in all animals were measured by ELISA to further evaluate the expression of inflammatory factors in chickens and rats with hyperglycemia induced by STZ (Table 8). The results showed that in rats with hyperglycemia, the concentrations of pro-inflammatory factors NF-κB p-p65 and IL-1β increased significantly, whereas the anti-inflammatory factor IL-10 decreased significantly. In contrast, in chickens, STZ-induced hyperglycemia led to non-significant upward trends in NF-κB p-p65 and IL-1β concentrations as well as a non-significant downward trend in IL-10.

### 3.7. Effect of STZ on Taurine and Other Free Amino Acids Concentration

We next sought to explore the cause for the fact that the concentration of FA and MG in chickens and rats increased significantly after STZ treatment, while the concentration of AGEs increased significantly in rats but did not increase significantly in chickens. The concentration of taurine and other free amino acids in chickens and rats was determined by LCMS. The results showed that in terms of interspecies differences, the taurine concentration in chickens was significantly higher than that in rats only in the control group (*p* < 0.05, Table 9). After STZ induction, the taurine concentration in chickens decreased significantly (*p* < 0.05), while there was no significant change in taurine content in rats. In addition, Table 9 also presented other significant differences in free amino acids between chickens and rats under different treatment conditions. For the other 16 free amino acids, except for glycine and phenylalanine, the concentrations of other free amino acids were all significantly reduced in chickens treated with STZ (*p* < 0.05; Table 9). However, in rats, after STZ induction, except for a significant decrease in glutamic acid, the concentrations of alanine, proline, valine, and phenylalanine were all significantly increased, and there was no significant difference in others.

## 4. Discussion

Poultry are unique among vertebrates in maintaining relatively high blood glucose concentrations. They live healthily without adverse effects despite high blood glucose concentrations compared with mammals [2,32]. Glycosylation of proteins and subsequent production of advanced glycation end products (AGEs) are the most important factors leading to hyperglycemia-induced pathological changes [33]. However, there are no studies investigating the relationship between the development of AGEs and potential side effects on chronic hyperglycemia in poultry. In this study, we compared the blood physiological differences between chickens and rats and assessed whether there were biomolecules in chicken blood that could prevent inflammation induced by streptozotocin (STZ)-induced hyperglycemia. We found that after STZ treatment, fasting blood glucose (FBG) levels in both chickens and rats increased significantly, but those in rats were higher than those in chickens. Moreover, STZ induction had no obvious effects on body weight, organ development, tissue damage, and inflammatory factor expression in chickens. In contrast, STZ-treated rats showed delayed body weight and organ development, and the expression levels of inflammatory factors were significantly increased in the target organs of hyperglycemia. For the expression of AGEs, chickens showed an upward trend but no significant change after STZ induction, while rats showed a significant increase. However, for the precursor substances of AGEs, fructosamine (FA) and methylglyoxal (MG), both chickens and rats showed increases after STZ treatment. The concentration of some free amino acids (including taurine), which inhibit the formation of AGEs, was significantly decreased in chickens, whereas disorder was observed in rats. Taken together, the main reason for the no deleterious effects exhibited with chronic hyperglycemia by chickens is that taurine and some free amino acids act as carbonyl scavengers to inhibit the formation of AGEs from FA and MG. In contrast, in hyperglycemic rats, free amino acids were not consumed, leading to the continuous accumulation of FA, MG, and even AGEs. Ultimately, inflammation occurred. This study revealed that taurine and other free amino acids in chickens act as carbonyl scavengers to inhibit the formation of AGEs, thereby providing a novel explanation for their resistance to hyperglycemic damage, which will provide new targets for developing therapeutic agents.

At present, chickens have been studied as potentially useful animal models in the case of diabetes mellitus [24,25]. For mammals, these chronically elevated concentrations of blood glucose lead over time to pathophysiological changes collectively known as diabetic complications, including retinopathy, nephropathy neuropathy, microvascular damage, and macrovascular damage [9,10]. However, there are no adverse effects on chickens, and the process in which substances in their blood are involved in blood glucose regulation remains unclear. Here, we first compared the factors that affect blood glucose concentrations of chickens and rats. Notably, consistent with previous results [34,35], the fasting blood glucose level of chickens was 1.5–2 times that of rats when both were in a normal physiological state and had no additional glucose intake. Chickens are an insulin-resistant species characterized by a low concentration of insulin and high level of blood glucose [2,20]. This is consistent with our results showing that in the oral glucose tolerance test (OGTT) and intraperitoneal insulin tolerance test (IPITT), as well as in the insulin and glucagon detection assays, chickens exhibit distinct insulin-resistant physiological characteristics that differ from those of rats. As a glucosamine–nitrosourea antibiotic, streptozotocin (STZ) induces specific damage to pancreatic β-cells [36,37]. Due to its chemical resemblance to glucose, STZ penetrates the cell membrane via glucose transporter 2 (GLUT2) after injection. The subsequent intracellular toxicity leads to irreversible necrosis and a severe reduction in insulin production, thus elevating the blood glucose levels [38,39]. Therefore, we induced hyperglycemia in chickens and rats by intraperitoneal injection of STZ and found that the blood glucose concentrations of both chickens and rats increased significantly, but those in rats were higher than those in chickens. The OGTT and IPITT values of STZ-induced rats were significantly higher than those of the STZ-induced chickens. However, there was no significant difference between STZ-induced chickens and the control group. Moreover, the concentrations of both insulin and glucagon in rats were significantly higher than those in chickens. From the results of STZ induction, we found that chickens exhibited blood glucose metabolic stability that differs from that of rats.

Advanced glycation end products (AGEs) are a series of irreversible products formed by carbonyl and amino groups through the Schiff base and the Amadori rearrangement, and their formation is accelerated in hyperglycemia [40]. This process is known as the Maillard reaction due to the associated yellow–brown color change [41]. Since both types of compounds are indispensable for life and have to coexist in cells and organisms, Maillard reactions are unavoidable and lead to the formation of AGEs that are invariably deleterious to cell and organism function [42,43,44]. One way by which AGEs exert their damaging effects is through interaction with specific receptors known as the receptor for advanced glycation end products (RAGEs), via which AGEs largely activate signaling mechanisms that cause cell stress, contribute to cellular dysfunction, and damage target organs, leading to complications [45,46,47]. Therefore, RAGEs are considered to be one of the important factors contributing to diabetic complications [24]. The engagement of AGEs with RAGEs activates a plethora of downstream signaling pathways, and the nuclear factor-kappa B (NF-κB) pathway is one of the central and pivotal inflammatory pathways [48,49]. Upon the activation of the RAGE, the phosphorylation and degradation of the inhibitory protein IκBα are induced, and the NF-κB dimer (primarily the p65/p50 heterodimer) is thereby released, enabling it to translocate into the nucleus [50]. Within the nucleus, NF-κB, particularly the phosphorylated active form of p65 (p-p65), serves as a master transcriptional switch, thereby triggering the expression of a wide array of pro-inflammatory cytokines and chemokines, including interleukin-1β (IL-1β), tumor necrosis factor-alpha (TNFα), and monocyte chemoattractant protein 1 (MCP1) [51,52]. This cascade creates a sustained pro-inflammatory microenvironment, which is a fundamental driver of the tissue damage and dysfunction observed in diabetic complications [53,54]. In this study, we assessed the activation status of this pathway by measuring the mRNA and protein levels of NF-κB total p65 (t-p65), NF-κB phosphorylated p65 (p-p65), and one of its key downstream targets, IL1β. We found that STZ-induced hyperglycemic rats exhibited a significant upregulation of AGEs, alongside elevated p-p65 and IL1β, strongly supporting the notion that the AGEs/RAGE-NF-κB axis is robustly activated, culminating in inflammatory damage. Conversely, the absence of significant AGE accumulation in chickens precludes the initiation of this deleterious signaling cascade, as evidenced by the stable levels of p-p65 and IL1β. Our findings provide a more upstream and critical explanation: AGEs themselves failed to accumulate in hyperglycemic chickens, which is the direct reason for the absence of inflammation. This contrasts sharply with the significant AGEs accumulation and subsequent inflammatory response in STZ-induced rats. Accordingly, it is more crucial to explore the reasons for maintaining the low concentration of AGEs in chicken blood.

Consequently, this study further detected the concentrations of fructosamine (FA) and methylglyoxal (MG), which are important precursor substances of AGEs [55]. FA is a product of the Schiff base reaction in the early stage of the Maillard reaction, referring to all ketoamine linkages that result from the glycation of serum proteins [17,18]. MG is an intermediate product of the Maillard reaction, having two adjacent carbonyl groups that can directly form AGEs [46,56]. We found that the concentration of FA in both chickens and rats with hyperglycemia was significantly higher than that in the control group. However, regarding the concentration of MG, there was no significant increase in hyperglycemic rats, while there was a significant increase in hyperglycemic chickens. For AGE concentrations, there was no significant difference in chickens between the two groups, while there was an extremely significant difference in rats. While both FA and MG increased significantly in hyperglycemic chickens, the final production of AGEs was effectively blocked. This critical disconnect between elevated precursors and stable AGE levels strongly suggests the presence of active scavenging substances in chicken blood that intercept the Maillard reaction pathway.

Our results demonstrated significantly higher basal levels of several free amino acids (e.g., taurine, arginine, histidine, and alanine) in chickens compared to rats. This finding is particularly intriguing in the context of their established role as carbonyl scavengers, which competitively inhibit the carbonyl-amine reactions that lead to AGE formation [12,28]. For example, it was found that taurine, as well as other free amino acids including arginine, leucine, and glycine, could inhibit the production of glycation products by reacting with aldehyde groups [28,29]. Moreover, free amino acids could directly further form dipeptides or polypeptides to enhance the effect of scavenging AGEs, such as homocarnosine [57]. Crucially, upon STZ induction, we observed a significant decrease in the concentrations of these specific amino acids specifically in chickens, concomitant with a successful blockade of AGEs accumulation. We interpret this dynamic consumption as indirect evidence of their active engagement in neutralizing reactive carbonyl species derived from glucose and MG, thereby explaining the absence of hyperglycemic complications. This stands in stark contrast to rats, which lacked both high basal levels and this consumption response, resulting in significant AGEs accumulation. While our study was not designed to establish direct causality—a limitation we acknowledge—this correlative evidence is bolstered by preliminary functional data. Our preliminary research results showed that intraperitoneal injection of arginine and taurine in STZ-induced hyperglycemic rats significantly reduced AGE concentrations [2]. This prior finding lends indirect support to the hypothesis that the higher availability and dynamic consumption of these amino acids in chickens constitute a key mechanism for their resistance to AGEs formation.

However, we recognize that our discussion attributes a primary protective role to free amino acids, while other factors—such as differences in mitochondrial metabolism or antioxidant systems—were not investigated and could also contribute to the observed phenotype [58,59,60]. Furthermore, our study operated on the premise that STZ induces β-cell damage in chickens via a mechanism analogous to that in mammals, yet direct evidence for GLUT2 transporter expression in chicken β-cells and quantitative histological confirmation of islet damage are lacking. Future studies employing taurine synthesis inhibition in chickens or amino acid supplementation in hyperglycemic models, coupled with measurements of key enzyme activities (e.g., glyoxalase I), as well as direct quantification of pancreatic islet morphology and GLUT2 expression, are essential to definitively confirm the causal role of these candidate amino acids and elucidate their relative importance among other potential protective mechanisms.

## 5. Conclusions

In summary, in contrast to rats, the key reason why chickens do not develop complications despite chronic hyperglycemia resides in the enrichment of taurine, alanine, and other free amino acids in their bloodstream. These amino acids function as endogenous carbonyl scavengers, effectively abrogating the formation of AGEs and precluding subsequent tissue damage mediated through inflammatory signaling pathways. In contrast, due to the low content and poor mobilization capacity of free amino acids in rats, these amino acids have failed to act as carbonyl scavengers to eliminate AGEs, leading to inflammation in the body. Our study affords novel insights into the absence of complications in poultry with chronic hyperglycemia and provides critical leads for the development of novel therapeutic strategies for diabetes mellitus.

## Figures and Tables

**Table 1:** Standard diet composition of chickens.

Ingredient	Purity/(%)	Major Nutrient Factors	Nutrient Level
Corn	56.75	ME/(MJ/kg)	13.22
Wheat	8.00	CP/(%)	18
Soybean meal	26.00	Ca/(%)	1.02
Fat soybean	0.00	AP/(%)	0.7
Corn protein	0.00	Lysine/(%)	1.1
Calcium hydro phosphate	1.00	Methionine/(%)	0.5
Mountain flour	1.25		
Soybean oil	5.00		
Fish meal	0.00		
Premix	2.00		
Total	100.00		

**Table 2:** Standard diet composition of rats.

Ingredient	Basic Diet	Total					
Gm/(%)	1	1					
Protein/(%)	18.27	18.27					
Fat/(%)	4.8	4.8					
Fiber/(%)	3.57	3.57					
Ash/(%)	5.89	5.89					
Calcium/(%)	1.22	1.22					
Phosphorus/(%)	0.839	0.839	Energetic substance	Protein	Fat	Carbohydrate	
			Gm/(%)	18.27	4.8	57.47	Total energy
			Kcal/(kg)	730.8	432	2298.8	3461.6
			Kcal/(%)	21.11	12.48	66.41	100

**Table 3:** Quantitative polymerase chain reaction primer sequences.

Name of Primer	Sequence/(5′-3′)	Accession Number	Amplicon Length/(bp)
c *NF-κB*-F	TTCTCCACTTGGCGATCATTCACG	NM_001001472.3	109
c *NF-κB*-R	GTCTGGCTGAGGTTGTTCTGGAAG		
c *IL-1β*-F	GCCTGCAGAAGAAGCCTCG	NM_204524.2	210
c *IL-1β*-R	GGAAGGTGACGGGCTCAAAA		
c *TNFα*-F	TGTGTATGTGCAGCAACCCGTAGT	XM_015294121.4	229
c *TNFα*-R	GGCATTGCAATTTGGACAGAAGT		
c *MCP1*-F	AGTACCTCCCAGGCACAAA	NM_204438.2	192
c *MCP1*-R	ATGCTTCTTCACCCAGTCTTC		
c *IL-10*-F	TCAATCCAGAGACGATGAACTTAA	NM_001004414.4	235
c *IL-10*-R	GCAGGTGAAGAAGCGGTGA		
c *β-actin*-F	CTGGCACCTAGCACAATGAA	NM_205518.2	109
c *β-actin*-R	ACATCTGCTGGAAGGTGGAC		
r *NF-κB*-F	TGTGGTGGAGGACTTGCTGAGG	NM_001276711.2	138
r *NF-κB*-R	AGTGCTGCCTTGCTGTTCTTGAG		
r *IL-1β*-F	CCTATGTCTTGCCCGTGGAG	NM_031512.2	118
r *IL-1β*-R	CACACACTAGCAGGTCGTCA		
r *TNFα*-F	GGAGGGAGAACAGCAACTCC	NM_012675.3	93
r *TNFα*-R	GCCAGTGTATGAGAGGGACG		
r *MCP1*-F	GAGTCGGCTGGAGAACTACAAG	NM_031530.1	227
r *MCP1*-R	GGGTCAAGTTCACATTCAAAGG		
r *IL-10*-F	GCAGGACTTTAAGGGTTACTTGGGT	NM_012854.2	177
r *IL-10*-R	AGAAATCGATGACAGCGTCGCA		
r *β-actin*-F	GACCTGTACGCCAACACAGT	NM_031144.3	129
r *β-actin*-R	CTCAGGAGGAGCAATGATCT		

**Table 4:** Gradient of mobile phase for LCMS of methylglyoxal (MG).

Time/(min)	Flow Velocity/(mL/min)	Mobile Phase A/(%)	Mobile Phase B/(%)
0.0	0.3	95	5
1.0	0.3	95	5
5.0	0.3	50	50
6.5	0.3	40	60
6.6	0.3	10	90
7.5	0.3	10	90

**Table 5:** Mobile phase gradients for LCMS of free amino acids.

Time/(min)	Flow Velocity/(mL/min)	Mobile Phase A/(%)	Mobile Phase B/(%)
0.0	1.0	100	0
14.0	1.0	85	15
29.0	1.0	66	34
30.0	1.0	0	100
37.0	1.0	0	100
38.0	1.0	100	0
45.0	1.0	100	0

**Table 6:** Serum biochemical parameters of chickens and rats in each group.

Group	CON-Chicken	STZ-Chicken	CON-Rat	STZ-Rat
ALT/(U/L)	1.60 ± 0.38	1.70 ± 0.32	34.73 ± 1.63 ^b^	80.83 ± 15.02 ^a^
AST/(U/L)	240.70 ± 22.47	274.93 ± 74.59	173.33 ± 2.31 ^a^	127.90 ± 6.06 ^b^
AST/ALT	164.13 ± 29.79	159.97 ± 20.32	4.37 ± 0.30 ^a^	1.97 ± 0.13 B ^b^
ALP/(U/L)	2956.67 ± 1437.34	1204.67 ± 136.60	64.33 ± 1.86 ^b^	230.67 ± 2.60 ^a^
TP/(g/L)	29.70 ± 2.22	25.17 ± 0.57	62.27 ± 0.99 ^a^	55.07 ± 4.45 ^b^
ALB/(g/L)	11.37 ± 1.23	10.53 ± 0.28	33.13 ± 1.18 ^a^	24.70 ± 0.15 ^b^
GLO/(g/L)	18.33 ± 1.17 ^a^	14.63 ± 0.28 ^b^	29.53 ± 0.62 ^b^	34.47 ± 0.62 ^a^
A/G	0.62 ± 0.05	0.72 ± 0.01	0.97 ± 0.03	0.73 ± 0.89

Note: The values used for *t*-test analysis are presented as means ± SE and the values used for the non-parametric test are presented as medians (interquartile range). CON-chicken, control group chicken; STZ-chicken, streptozotocin (STZ)-treated group chicken; CON-rat, control group rat; STZ-rat, STZ-treated group rat. N = 6. ^a^, ^b^, *p* < 0.05.

**Table 7:** Indexes of blood lipids of chickens and rats in each group.

Group	CON-Chicken	STZ-Chicken	CON-Rat	STZ-Rat
TG/(mmol/L)	0.94 ± 0.24	0.53 ± 0.19	1.54 ± 0.23 ^a^	0.74 ± 0.12 ^b^
TC/(mmol/L)	2.80 ± 0.32	2.80 ± 0.18	1.35 ± 0.77	1.55 ± 0.39
LDL-C/(mmol/L)	2.08 ± 0.31	2.20 ± 0.21	0.40 ± 0.03	0.91 ± 0.40
HDL-C/(mmol/L)	0.76 ± 0.08	0.72 ± 0.04	0.59 ± 0.04	0.75 ± 0.21

Note: The values used for *t*-test analysis are presented as means ± SE and the values used for the non-parametric test are presented as medians (interquartile range). CON-chicken, control group chicken; STZ-chicken, streptozotocin (STZ)-treated group chicken; CON-rat, control group rat; STZ-rat, STZ-treated group rat. N = 6. ^a^, ^b^, *p* < 0.05.

**Table 8:** Comparison of different inflammatory factors concentrations in the serum of chickens and rats by ELISA.

Group	CON-Chicken	STZ-Chicken	CON-Rat	STZ-Rat
p-NF-κB p65/(pg/mL)	12.59 ± 0.63	14.11 ± 0.37	12.29 ± 0.33 ^b^	16.52 ± 0.30 ^a^
IL-1β/(pg/mL)	5.80 ± 0.67	7.56 ± 1.55	24.56 ± 2.10 ^b^	32.54 ± 1.91 ^a^
IL-10/(pg/mL)	19.35 ± 2.07	14.03 ± 1.69	27.27 ± 4.67 ^a^	9.35 ± 1.82 ^b^

Note: The values used for *t*-test analysis are presented as means ± SE and the values used for the non-parametric test are presented as medians (interquartile range). CON-chicken, control group chicken; STZ-chicken, streptozotocin (STZ)-treated group chicken; CON-rat, control group rat; STZ-rat, STZ-treated group rat. N = 6. ^a^, ^b^, *p* < 0.05.

**Table 9:** Comparison of different free amino acid contents in chickens and rats by LCMS.

Amino Acid	CON-Chicken	STZ-Chicken	CON-Rat	STZ-Rat
Tau/(mg/L)	37.596 ± 5.941 ^Aa^	15.758 ± 1.081 ^b^	20.773 ± 0.549 ^B^	19.248 ± 0.419
Asp/(mg/L)	7.195 ± 0.613 ^a^	4.351 ± 0.183 ^b^	6.315 ± 0.871	7.092 ± 0.727
Glu/(mg/L)	26.532 ± 4.039 ^Aa^	9.557 ± 0.212 ^b^	16.318 ± 0.659 ^Ba^	11.828 ± 0.189 ^b^
Ser/(mg/L)	11.083 ± 1.073 ^Ba^	6.352 ± 0.207 ^b^	22.277 ± 2.173 ^A^	19.450 ± 0.776
Gly/(mg/L)	11.125 ± 1.231 ^B^	9.408 ± 0.340	20.748 ± 1.369 ^A^	20.302 ± 0.413
His/(mg/L)	10.215 ± 1.982 ^Aa^	4.067 ± 0.456 ^b^	4.188 ± 0.268 ^B^	4.845 ± 0.314
Arg/(mg/L)	16.597 ± 2.661 ^Aa^	7.143 ± 1.292 ^b^	13.965 ± 1.539 ^B^	13.215 ± 0.260
Thr/(mg/L)	13.797 ± 2.340 ^Aa^	5.957 ± 0.375 ^b^	9.860 ± 2.400 ^B^	7.560 ± 0.668
Ala/(mg/L)	33.442 ± 4.475 ^Aa^	15.557 ± 0.966 ^b^	23.547 ± 0.844 ^Bb^	28.013 ± 0.725 ^a^
Pro/(mg/L)	19.075 ± 0.646 ^Ba^	13.667 ± 0.080 ^b^	27.178 ± 2.112 ^Bb^	38.473 ± 1.289 ^a^
Tyr/(mg/L)	8.425 ± 0.866 ^Ba^	4.322 ± 0.261 ^b^	13.502 ± 1.877 ^A^	15.640 ± 1.544
Val/(mg/L)	14.908 ± 0.640 ^Aa^	12.105 ± 0.895 ^b^	7.575 ± 0.618 ^Bb^	9.062 ± 0.723 ^a^
Met/(mg/L)	4.202 ± 0.437 ^Aa^	1.740 ± 0.103 ^b^	1.487 ± 0.699 ^B^	1.672 ± 0.049
Ile/(mg/L)	9.288 ± 0.864 ^Aa^	6.042 ± 0.130 ^b^	2.538 ± 0.248 ^B^	2.682 ± 0.148
Leu/(mg/L)	10.267 ± 0.86 ^Aa^	6.692 ± 0.242 ^b^	6.365 ± 0.369 ^B^	7.388 ± 0.293
Phe/(mg/L)	39.827 ± 0.330 ^A^	42.248 ± 5.973	33.187 ± 0.326 ^Bb^	36.522 ± 0.386 ^a^
Lys/(mg/L)	17.708 ± 3.813 ^Aa^	5.127 ± 0.077 ^b^	1.488 ± 0.251 ^B^	1.255 ± 0.054

Note: The values used for *t*-test analysis are presented as means ± SE and the values used for the non-parametric test are presented as medians (interquartile range). CON-chicken, control group chicken; STZ-chicken, streptozotocin (STZ)-treated group chicken; CON-rat, control group rat; STZ-rat, STZ-treated group rat. N = 6. ^a^, ^b^, *p* < 0.05 vs. control group; ^A^, ^B^, *p* < 0.05 vs. chicken.

## Data Availability

All data generated or analyzed during this study are included in this published paper.

## Supplementary Materials

The following supporting information can be downloaded at: https://www.mdpi.com/article/10.3390/biology14121657/s1, Figure S1. Effects of streptozotocin (STZ) on the expression of inflammatory marker proteins in chicken tissues. Figure S2. Effects of streptozotocin (STZ) on the expression of inflammatory marker proteins in rat tissues.
