# Supplementary material for: Inhibition of Advanced Glycation End Products: A Nexus of Chicken Hyperglycemia and Inflammation Absence
Source: Biology (Basel). 2025 Nov 24;14(12):1657. doi: 10.3390/biology14121657 (PMC12730405; doi:10.3390/biology14121657)
Supplement: Supplementary file 1 [file biology-14-01657-s001.zip › biology-3974253-supplementary.pdf]

Supplementary Data

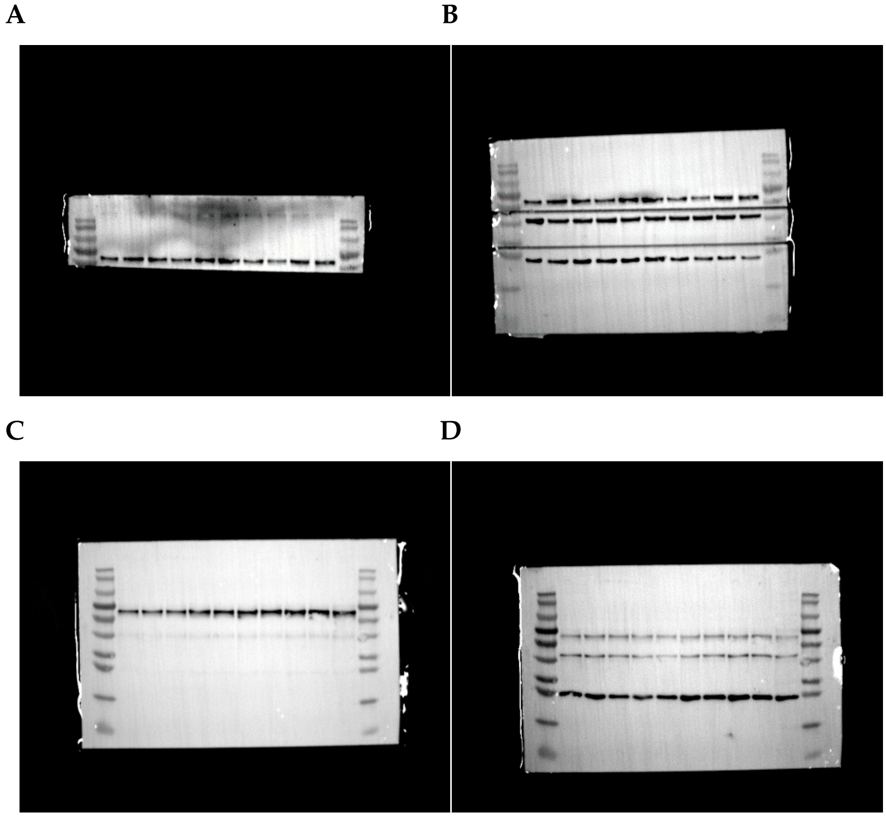

Figure S1. Effects of streptozotocin (STZ) on the expression of inflammatory marker proteins in chicken tissues. (A-B) and (C-D) represent two independent biological replicates (N=2 per group). (A) Protein levels of total NF-κB p65 in chicken tissues before and after STZ treatment were determined by Western blot using cropped PVDF membranes. (B) Protein levels of p-NF-κB p65, β-actin, and IL-1β in chicken tissues before and after STZ treatment were determined by Western blot using cropped PVDF membranes. (C) Protein levels of total NF-κB p65 in chicken tissues before and after STZ treatment were determined by Western blot using uncropped PVDF membranes. (D) Protein levels of p-NF-κB p65, β-actin, and IL-1β in chicken tissues before and after STZ treatment were determined by Western blot using uncropped PVDF membranes.

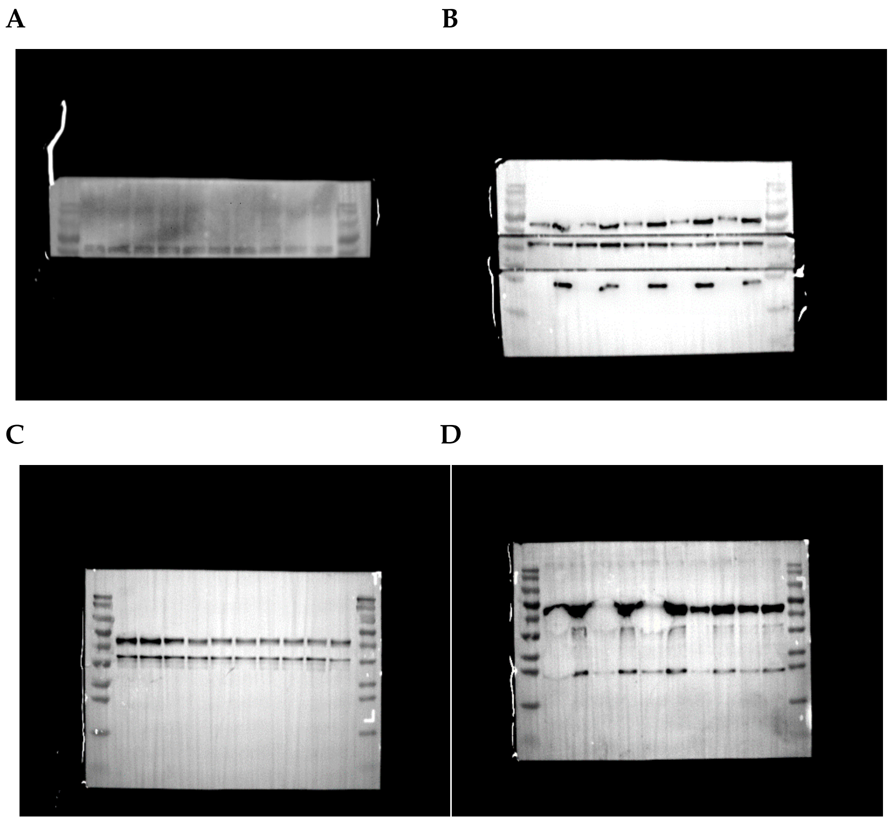

Figure S2. Effects of streptozotocin (STZ) on the expression of inflammatory marker proteins in rat tissues. (A-B) and (C-D) represent two independent biological replicates (N=2 per group). (A) Protein levels of total NF-κB p65 in rat tissues before and after STZ treatment were determined by Western blot using cropped PVDF membranes. (B) Protein levels of p-NF-κB p65, β-actin, and IL-1β in rat tissues before and after STZ treatment were determined by Western blot using cropped PVDF membranes. (C) Protein levels of total NF-κB p65 and β-actin in rat tissues before and after STZ treatment were determined by Western blot using uncropped PVDF membranes. (D) Protein levels of p-NF-κB p65 and IL-1β in rat tissues before and after STZ treatment were determined by Western blot using uncropped PVDF membranes.
